# A Department of Public Safety

**Published:** 1890-10-25

**Authors:** 


### A Department of Public Safety.

All large cities in civilized countries now have, as a part of their municipal organization, an officer or officers whose duties relate to the preservation of the public health; but the duties and powers of these officers and their relations to other departments of the public service vary greatly, and nowhere are these differences greater than in the large cities of the United States. Some of them, such as Boston and New York, have boards of health; others have health officers; in some the department of health has charge of the registration of births and deaths, of the cleansing of streets and alleys, of the removal of garbage, and of the supervision of house drainage; while in others it has nothing to do with many, or in some cases with any, of these subjects.

As a specimen of an unusual form of organization we may take the Department of Public Safety of the City of Pittsburgh, in the State of Pennsylvania, a copy of the second annual report of which, for the year 1889, is now before us. The executive powers of this city are vested in the mayor and in five departments, viz.: a Department of Public Safety, a Department of Public Works, a Department of Charities, a Department of Awards, and a Department of Law. The Department of Public Safety includes all matters relating to the public health, to the fire and police force, the city telegraphs, and the inspection of buildings, including plumbing, gasfitting, and house drainage, and is under the direction of one person, known as the "Chief of the Department of Public Safety." Under his supervision is the Bureau of Health, which consists of a superintendent, with clerks and inspectors. The relative size and importance of the different bureaux may be estimated somewhat from the annual appropriations, which for the last year were as follows:—

| | Total Appropriation. | Appropriation for Salaries. |
|---|---|---|
| General Office ... ... ... | $10,100 ... | $10,000.00 |
| Bureau of Fire ... ... | 249,000 ... | 174,208.92 |
| Bureau of Police ... ... | 309,000 ... | 247,650 00 |
| Bureau of Electricity ... ... | 40,000 ... | 12,990.36 |
| Bureau of Health ... ... | 39,500 ... | 18,577.50 |
| Bureau of Building Inspection | 4,825 ... | 4,181.50 |
| Bureau of Plumbing and House Drainage ... ... ... | 2,075 ... | 1,500.00 |

The superintendent of the Bureau of Health is, apparently, not a medical man, but he has a physician as Registrar of Vital Statistics, and also three inspectors, ten sanitary policemen, two clerks, and six employés.

Pittsburgh is a city containing about 238,500 inhabitants, as shown by the census of the 1st of June last; is devoted to manufactures, and especially to iron and steel works; and is famous for the extensive use of natural gas which is there utilized not only for lighting, but for cooking, heating, and the production of steam-power. Its growth has been rapid, and seems to have outstripped the provisions made for preserving the public health. The water supply of that part of the city known as the south side and east-end districts is more or less polluted, and at least one outbreak of fever has been traced to this cause. The sewerage and drainage of the city are incomplete and unsatisfactory; but no definite information on this subject is given in this report, since it is under the control of an entirely different department. There is one furnace, worked by natural gas, for the cremation of garbage; but this is quite insufficient to dispose of all this material, and the consequence is that it is thrown at night into vacant lots, alleys, etc., for it would seem that the city does not itself manage the garbage-collecting service.

A curious note is to the effect that several hundred cesspools, with the waste water and filth from as many premises, are being drained into some abandoned coal mines which underlie a portion of the city, and that unpleasant odours are already perceptible as the result. The death-rate of the city was not high, being between 18 and 19 per 1,000; but of the 4,286 deaths reported, 213 were due to diphtheria, 218 to typhoid fever, and 245 to diarrhœal diseases.

The name "Department of Public Safety" is a very good one for educational purposes, and the bureaux placed under it are very properly associated together; but it would seem as if the bureau having charge of sewer construction might with advantage be classed as pertaining to the public safety. It is true that it is usual to place the construction and maintenance of sewers in the hands of the department having charge of the streets, and thus to entirely separate it from the subject of house drainage and plumbing inspection; but the two subjects are so closely connected that in many respects it would save time and money if they were inspected and managed by the same officials.

